# Decidualization of Stromal Cells Promotes Involvement of Mast Cells in Successful Human Pregnancy by Increasing Stem Cell Factor Expression

**DOI:** 10.3389/fimmu.2022.779574

**Published:** 2022-01-31

**Authors:** Chiyuki Ueshima, Tatsuki R. Kataoka, Mitsumasa Osakabe, Akihiko Sugimoto, Akihiko Ushirokawa, Yuji Shibata, Hiroya Nakamura, Rintaro Shibuya, Sachiko Minamiguchi, Tamotsu Sugai, Hironori Haga

**Affiliations:** ^1^Department of Diagnostic Pathology, Kyoto University Hospital, Kyoto, Japan; ^2^Department of Pathology, Iwate Medical University, Yahaba-cho, Japan; ^3^Department of Molecular Diagnostic Pathology, Iwate Medical University, Yahaba-cho, Japan; ^4^Department of Dermatology, Kyoto University Hospital, Kyoto, Japan

**Keywords:** decidual stromal cell, leukemia inhibitory factor (LIF), mast cell, pregnancy, stem cell factor (SCF), trophoblast

## Abstract

Decidualization of endometrial stromal cells and the presence of immunocompetent cells, including human mast cells, play important roles in the establishment of pregnancy. In the present study, the effects of decidualization of endometrial stromal cells on the function of decidual mast cells were elucidated. The *in vitro* assay revealed that decidualization of an endometrial stromal cell line, T HESCs, increased stem cell factor (SCF) mRNA expression. Decidualization of T HESCs enhanced the production of leukemia inhibitory factor (LIF), and the migration of LAD2 cells when co-cultured with T HESCs and LAD2 cells. In addition, decidualization of T HESCs enhanced cell migration in a human trophoblast cell line, HTR-8/SVneo, increased CD9 expression, a marker for extravillous trophoblast (EVT) differentiation, and decreased the secretion of β human chorionic gonadotropin (hCG), a marker for syncytiotrophoblast (ST) differentiation, when co-cultured with T HESCs, LAD2 cells, and HTR-8/SVneo cells, in a LIF-dependent manner. Histological samples from uterine pregnancies, including decidual stromal cells, showed increased SCF mRNA expression, mast cell numbers and LIF mRNA expression thereof compared with tubal pregnancy. SCF produced by decidual stromal cells enhanced the migration and LIF production of mast cells, and promoted the migration and differentiation of trophoblasts to increase the likelihood of successful human pregnancy.

## Introduction

Decidual immunocompetent cells are believed to be necessary for establishing pregnancy (1). Decidual natural killer (NK) cells secrete angiogenic factors, such as vascular endothelial growth factor (VEGF), angiopoietin-2, placental growth factor, and chymase. This is followed by angiogenesis and spiral artery remodeling in decidual tissues (2, 3). Recently, a new subset of decidual NK cells called pregnancy-trained decidual NK cells (PTdNKs), have been characterized as enhancers of proper placentation (4). Other types of immunocompetent cells important for establishing pregnancy include decidual regulatory T cells, which induce tolerance in the fetus *via* semi-allogenic grafts (5). Recently, decidual mast cells have also received attention regarding their involvement in the establishment of pregnancy (6).

Mast cells are immunocompetent cells (7) present in almost all mouse and human tissues, including decidual tissue (8). Analysis of mast cell-deficient *KIT^W-sh/W-sh^* mice showed that mast cells are dispensable, but enhance the establishment of pregnancy (9, 10). Mast cell chymase (MCC) is considered necessary for decidual vascular remodeling in mice and humans (3). We previously reported that human mast cells express killer cell immunoglobulin-like receptor 2DL4 (KIR2DL4/CD158d), a member of the KIRs that plays an important role in human pregnancy by inducing the secretion of leukemia inhibitory factor (LIF) (11, 12).

LIF is a member of the interleukin (IL)-6 family of cytokines. The LIF receptor consists of gp130 and a LIF receptor β subunit. The LIF receptor also transduces Janus kinase–signal transducer and activator of transcription (STAT) signaling (13). LIF plays important roles in establishing pregnancy (14). Female mice with LIF-knockout or defective gp130-mediated STAT signaling are infertile. This is owed to embryo implantation failure (15, 16). LIF is highly expressed in the endometrial glands, as well as decidual NK and mast cells (12, 14, 17).

LIF plays an important role in the differentiation of cytotrophoblasts into extravillous trophoblasts (EVTs) and syncytiotrophoblasts (STs). EVTs anchor the placenta to the uterine wall and are involved in maternal spiral artery remodeling. STs secrete hormones necessary for pregnancy maintenance and form the primary interface between maternal and fetal tissue, which facilitates nutrient and gas exchange. LIF is known to increase EVT differentiation and decrease ST differentiation (18).

Decidual stromal cells are also necessary for establishing pregnancy (19). Decidualization refers to the process of transformation of endometrial stromal cells into specialized secretory cells (decidual stromal cells), both morphologically and functionally. Endometrial stromal cells are elongated fibroblast-like cells. In contrast, decidual stromal cells are enlarged, round-shaped cells with larger nuclei and abundant cytoplasm. After decidualization, stromal cells produce new cellular products, including prolactin (PRL) (19). The decidual process requires elevated intracellular cAMP levels and sustained activation of the protein kinase A pathway, in addition to progesterone (19). Decidual stromal cells interact with decidual immunocompetent cells to establish a successful pregnancy (19). The indispensable role of decidualization was demonstrated in IL-11- or IL-11 receptor α−deficient mice. These mice had a defect in decidualization, which resulted in infertility (20, 21).

Decidual NK cells induce IL-15 production from decidual stromal cells followed by proliferation and recruitment into decidual tissues of NK cells (22). Decidual stromal cells reportedly inhibit NK cell cytolytic activity and IFN-γ production. This also inhibits the differentiation of dendritic cells, which can induce allogeneic T cell proliferation (23). Interactions between decidual stromal cells and decidual macrophages or NK cells are thought to enhance trophoblast invasion by some growth factors and cytokines (24). The association between decidual mast cells and decidual stromal cells has not been fully established.

In the present study, the association between the decidualization of endometrial stromal cells and mast cells in human pregnancy was investigated.

## Materials and Methods

### Patients

Histological samples were obtained from Kyoto University Hospital and Iwate Medical University from 2013–2019. Tubal specimens derived from 22 patients with tubal pregnancy were included. Six patients were excluded from the evaluation because their samples did not include implantation sites or mRNA could not be extracted for real-time PCR analysis. The remaining 16 tubal specimens were designated as “Tubal pregnancy” samples (**Table 1**). We selected 19 endometrial curettage specimens (clinically classified as abortion) derived from age- and gestational age-matched patients who had given birth to multiple children (“Uterine pregnancy”) that included implantation sites, and three hysterectomy samples from patients diagnosed with placenta accreta (**Table 1**). The patients at Kyoto University Hospital signed the “Kyoto University Hospital Informed Consent Form for the Non-therapeutic Use of Histopathological Materials,” and the signed forms were uploaded into the electronic health records. This study was also approved by the Ethical Research Committee of Iwate Medical University (MH2021-096).

**Table 1:** Characteristics of the study subjects.

	Uterine pregnancy (n = 19)	Tubal pregnancy (n = 16)
Age (years)	36.8 ± 4.35 (range: 29–43)	36.6 ± 3.00 (range: 31–42)
Gestational weeks	8.00 ± 1.05 (range: 6–9)	6.8 ± 1.04 (range: 6–9)

### Cells

LAD2 cells (kindly provided by Dr. Kirschbaum, passages 3 - 10) were cultured in StemPro-34 containing recombinant human stem cell factor (SCF; Peprotech, Rocky Hill, NJ, USA) (25). HTR-8/SVneo cells (kindly provided by Dr. Graham, passage 3 - 12) (26) and T HESCs (purchased from the American Type Culture Collection; Manassas, VA, USA, passages 3 - 10) were cultured in RPMI1640 medium supplemented with 10% fetal bovine serum, 2 mM L-glutamine, 100 units/mL penicillin, and 100 μg/mL streptomycin. Decidualization of T HESCs was induced by culturing with 1 µM medroxyprogesterone acetate (MPA; #M1629; Sigma-Aldrich, St. Louis, MO, USA) and 0.5 mM 8-bromoadenosine 3’:5’-cyclic monophosphate (8-Br cAMP; #B7880; Sigma) for 5–6 days (27). We confirmed the decidualization of T HESCs based on the presence of morphological changes and increased PRL mRNA.

### Antibodies and Reagents

Anti-mast cell tryptase (MCT) antibody (mouse monoclonal IgG, Clone AA1) was purchased from Abcam (Cambridge, UK). Anti-human LIF antibody (goat polyclonal IgG, P15018) for neutralization assay was purchased from R&D Systems (Minneapolis, MN, USA). The control IgG was obtained from BD Biosciences (San Jose, CA, USA). Toluidine blue O was purchased from Merck Millipore (115930, Darmstadt, Germany).

### Real-Time PCR

To evaluate SCF mRNA levels, tissues were macrodissected from histological specimens and mRNA was extracted from each sample using an RNeasy FFPE kit (QIAGEN, Valencia, CA, USA). A total of 5 × 10^6^ decidualized or non-decidualized T HESCs were collected, and mRNA was extracted using the RNeasy Plus Mini Kit (QIAGEN). To evaluate CD9 mRNA levels, a total of 5 × 10^6^ HTR-8/SVneo cells from decidualized T HESC supernatants (alone or pre-co-cultured with 1 × 10^4^ LAD2 cells with control IgG or anti-LIF antibody for 48 h) were collected, and mRNA was extracted using the RNeasy Plus Mini Kit (QIAGEN). To confirm decidualization, a total of 5 × 10^6^ T HESCs were treated with MPA and 8-Br cAMP for 5–6 days. The cells were then collected and mRNA was extracted using the RNeasy Plus Mini Kit (QIAGEN). A total of 500 ng of each mRNA sample was used in the reverse transcription PCR (PrimeScript RT Master Mix; TaKaRa, Ohtsu, Japan). The reverse transcription PCR primers for SCF, CD9, and GAPDH were designed by and purchased from TaKaRa. The PCR primers for PRL (forward primer: 5’ TCATCTGGTCACGGAAGTACGT 3’; reverse primer: 5’ GCCCTCTAGAAGCCGTTTGG 3’) were also purchased from TaKaRa (27). PCR amplification was performed with using SYBR Premix Ex Taq and the Thermal Cycler Dice Real Time System II (TaKaRa), programmed with the following cycles: initial denaturation at 95°C for 30 s; PCR amplification (55 cycles) of 5 s at 95°C (denature), 10 s at 58°C (anneal), and 15 s at 72°C (extension); and a subsequent standard dissociation protocol. The expression levels of SCF, CD9, and PRL were normalized using GAPDH as a standard.

### Migration Assessment

Migration of LAD2 cells toward T HESCs cells was assessed using Transwell polycarbonate membranes without Matrigel coating (8-μm pores; BD Biosciences, Franklin Lakes, NJ, USA). Aliquots (400 μL) of StemPro-34 containing 5 × 10^4^ LAD2 cells were added to the upper chamber, with inserts in the lower chamber containing culture medium only, decidualized T HESCs or non-decidualized T HESCs cells grown to confluence. After an 8-h incubation, cells that migrated into the lower wells were collected and counted.

Migration of HTR-8/SVneo cells toward T HESCs with or without LAD2 cells was assessed using Matrigel-coated Transwell polycarbonate membranes (8 μm pores; BD Biosciences). Aliquots (400 μL) of cytokine-free StemPro-34 only or cytokine-free StemPro-34 containing 5 × 10^4^ LAD2 were incubated in the lower chamber containing no cells or decidualized T HESCs or non-decidualized T HESCs cells grown to confluence at 37°C. Then, 2.5 × 10^3^ HTR-8/SVneo cells in 100 μL of cytokine-free medium were added to the upper chamber with control IgG or anti-LIF neutralizing antibody. After an 18-h incubation, the cells that migrated to the bottom of the upper wells were fixed with 4% paraformaldehyde, stained with Diff-Quick stain (Sysmex, Kobe, Japan) and counted under a microscope.

### ELISA for LIF and β Human Chorionic Gonadotropin (hCG)

For the LIF assay, LAD2 cells were cultured for 8 h in 200 μL of cytokine-free StemPro-34 only, cytokine-free StemPro-34 with 100 ng/mL SCF, and cytokine-free StemPro-34 with non-decidualized or decidualized T HESCs (1 × 10^5^). Aliquots (100 μL) of the supernatants were collected and used in ELISA kits (Human LIF ELISA Kit; ab100582; Abcam) according to the manufacturer’s protocol.

For the β hCG assay, decidualized T HESCs were cultured with cytokine-free StemPro-34 only or with LAD2 administrated with control IgG or anti-LIF antibody for 24 h. The culture supernatants (200 μL) including LAD2 cells were added to 5 × 10^4^ HTR-8/SVneo cells. After a 24-h incubation, aliquots (100 μl) of the supernatants were collected. In addition, aliquots (100 μL) of the supernatants were also collected from 5 × 10^4^ HTR-8/SVneo cells cultured with 20 μM forskolin (FK; Calbiochem, San Diego, CA, USA) in 200 μL of cytokine-free StemPro-34 for 48 h. The aliquots were used in ELISA kits (Free beta-CG (Human) ELISA Kit; #KA0210; Abnova, Taipei, Taiwan) according to the manufacturer’s protocol.

### Toluidine Blue Staining, Immunohistochemistry and RNA *In Situ* Hybridization

To count the number of mast cells in tissues, toluidine blue staining and immunohistochemistry with anti-MCT were utilized. To perform toluidine blue staining, tissue sections were deparaffinized with xylene, rehydrated, and incubated for 2–3 min in a solution of toluidine blue O (1 g) dissolved in 70% isopropanol (100 ml). After washing in distilled water, tissues were dehydrated with 90% ethanol (EtOH) and 100% EtOH. Tissues were then mounted on slides. To perform MCT immunostaining, tissue sections were deparaffinized with xylene, rehydrated, and pretreated with 0.3% hydrogen peroxide for 5 min. After steam heating for 30 min, anti-MCT antibody was added as the primary antibody (1:300 dilution), and the sections were incubated for 90 min at room temperature following blockade of background staining using Protein Block (X0909; DakoCytomation, Glostrup, Denmark). Staining was performed using the ultraView universal alkaline phosphatase red detection kit (Roche, Basel, Switzerland) according to the manufacturer’s instructions. Sections were imaged under a BX45 microscope equipped with a DP26 digital camera (Olympus, Tokyo, Japan). The top three fields (×200) where toluidine blue-stained or MCT-positive cells present in decidual tissue were selected, and the total cell number in each sample was counted, as described in our previous report (12).

To detect LIF mRNA-producing mast cells, RNA *in situ* hybridization (RNA-ISH) for LIF mRNA and MCT immunostaining was performed on the same sections. First, RNA-ISH was performed using RNAscope Target Probe Hs-LIF RNAscope 2.5 HD Detection Reagent-Brown (Advanced Cell Diagnostics, Hayward, CA, USA) according to the manufacturer’s instructions without counterstaining. The sections were then incubated with anti-MCT (1:300 dilution) for 30 min and positive signals were visualized with the ultraView universal alkaline phosphatase red detection kit (Roche). Nuclei were stained with hematoxylin.

### Statistical Analysis

**Figure 1:**
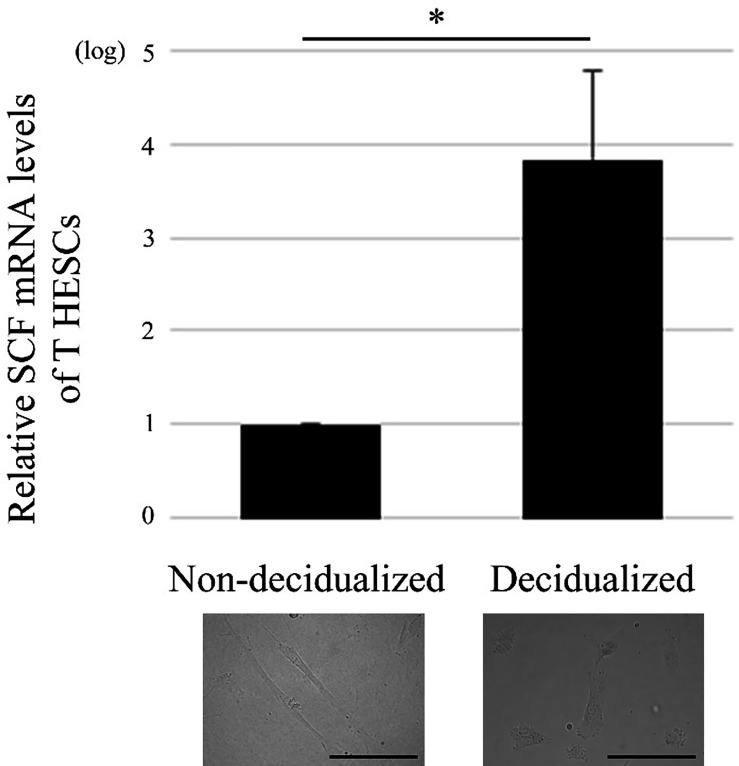
Decidualization of the human endometrial stromal cell line, T HESCs, increases SCF mRNA expression. Real time PCR was performed using mRNA extracted from decidualized or non-decidualized T HESCs (n = 3). *P < 0.05 compared with non-decidualized T HESCs. Bars = 50 μM.

**Figure 2:**
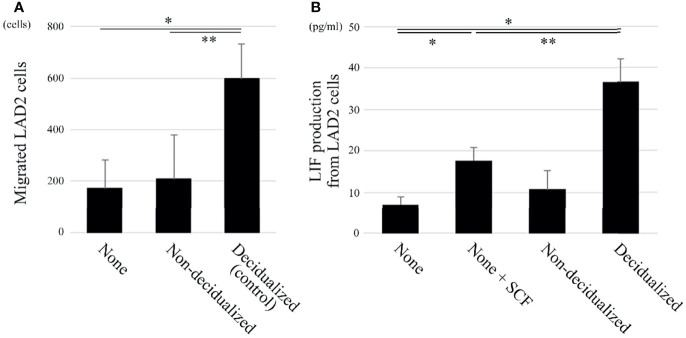
The migration and LIF production of the human mast cell line LAD2 are increased when co-cultured with decidualized T HESCs compared with non-decidualized T HESCs. **(A)** Two-chamber assay (n = 3). The upper chambers containing LAD2 cells were inserted into the lower chambers, which contained culture medium only (“none”), or decidualized or non-decidualized T HESCs cells. After an 8-hour incubation, the cells migrating into the lower chambers were counted. *P < 0.05 compared with none and **P < 0.05 compared with non-decidualized T HESCs in the lower chamber. **(B)** LIF ELISA (n = 5). LAD2 cells were cultured in cytokine-free medium only (“none”), medium with SCF (“none + SCF”), and cytokine-free medium with non-decidualized or decidualized T HESCs for 8 (h) The supernatants were collected and used in ELISA kits. *P < 0.05 compared with none and **P < 0.05 compared with none + SCF.

**Figure 3:**
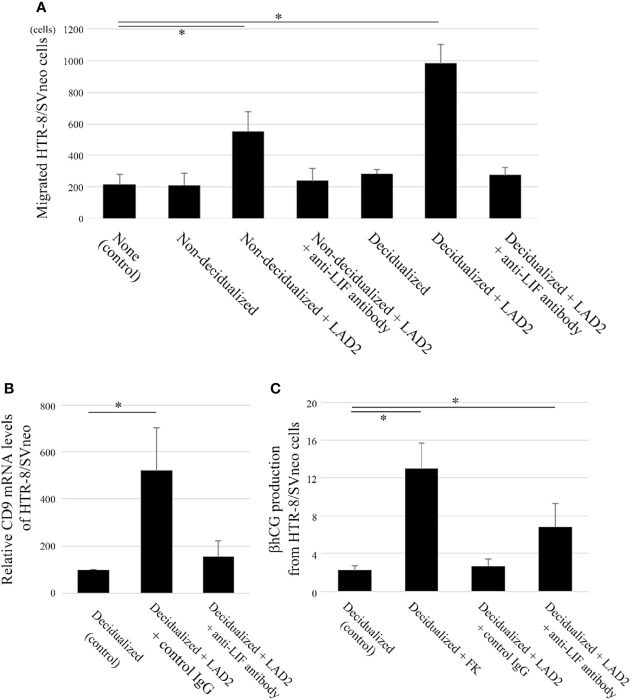
LIF derived from LAD2 increases migration and extravillous trophoblast (EVT) differentiation (based on CD9 expression) and decreases syncytiotrophoblast (ST) differentiation (based on β human chorionic gonadotropin [hCG] expression) in the human trophoblast cell line HTR-8/SVneo when co-cultured with decidualized T HESCs. **(A)** Two-chamber assay (n = 5). The upper chambers containing HTR-8/SVneo cells with control IgG or anti-LIF neutralizing antibodies were inserted into the lower chambers containing culture medium only (“none”), decidualized T HESCs or non-decidualized T HESCs cells with or without LAD2. After an 18-hour incubation, the cells that migrated to the bottom of the upper wells were counted. *P < 0.05 compared with none. **(B)** CD9 mRNA (n = 4). HTR-8/SVneo cells with culture supernatants of decidualized T HESCs (alone or pre-co-cultured with LAD2 cells with control IgG or anti-LIF antibody for 48 h) were collected, and mRNA was extracted and utilized for real time PCR. *P < 0.05 compared with decidualized T HESCs. **(C)** β hCG ELISA (n = 4). The culture supernatants from decidualized T HESCs incubated with cytokine-free medium only, or with LAD2 and control IgG or anti-LIF antibody were added to HTR-8/SVneo cells. After a 24-h incubation, the supernatants were collected (“Decidualized”, “Decidualized + LAD2 + control IgG”, or “Decidualized + LAD2 + anti-LIF antibody”). In addition, the supernatants from HTR-8/SVneo cells cultured with forskolin (FK) for 48 h (“Decidualized + FK”) were also collected. *P < 0.05 compared with Decidualized.

**Figure 4:**
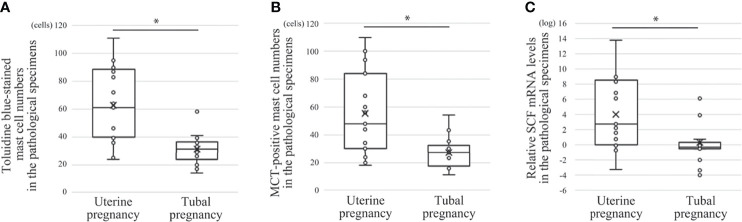
Comparison of mast cell numbers and SCF mRNA between histological specimens from uterine and tubal pregnancy (19 cases of uterine pregnancy *vs.* 16 of tubal pregnancy). **(A)** Toluidine blue-stained mast cell numbers. *P < 0.05 compared with uterine pregnancy. **(B)** MCT-positive mast cell numbers. *P < 0.05 compared with uterine pregnancy. **(C)** Relative SCF mRNA levels. Real time PCR was performed using mRNA extracted from the macrodissected histological specimens. *P < 0.05 compared with uterine pregnancy.

**Figure 5:**
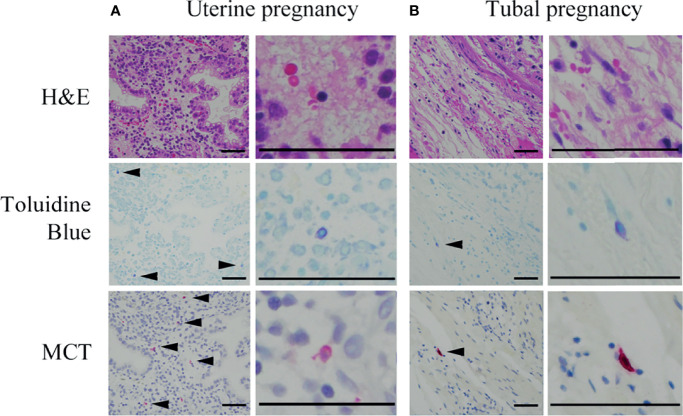
Representative photographs of mast cells in histological specimens from uterine and tubal pregnancies. Mast cells were detected using toluidine blue (as metachromatic cells [arrowheads]) and MCT immunostaining (red [arrowheads]). Bars = 100 μM.

In **Figures 1**–**5**, data are expressed as means ± standard error (SE). Differences between groups were examined for statistical significance using Student’s *t*-test (**Figure 1**), the Steel-Dwass multiple comparison test (**Figure 2**), Steel’s multiple comparison test (**Figure 3**), or unpaired Student’s *t*-test (**Figures 4**, **5**) (Excel [Office 2019]; Microsoft Corp., Redmond, WA, USA and HAD [https://norimune.net/had]). In all analyses, *P* < 0.05 was considered statistically significant.

## Results

### Decidualization Increases SCF mRNA Level in T HESCs

First, the phenotype of T HESCs, which can influence mast cells, was examined. SCF is an important cytokine in mast cell biology (7), so the SCF mRNA levels in T HESCs were evaluated. Decidualization increased SCF mRNA level in T HESCs (**Figure 1**).

### Migration and LIF Production of LAD2 Cells Are Increased When Co-Cultured With Decidualized T HESCs Compared With Non-Decidualized T HESCs

SCF induces the migration of mast cells (7). The migration of LAD2 cells to T HESCs was evaluated using a two-chamber assay. The migration of LAD2 cells toward decidualized T HESCs was enhanced compared with that toward non-decidualized T HESCs or cytokine-free culture medium only (**Figure 2A**).

LIF plays important roles in the establishment of uterine pregnancy (14), and we previously showed that decidual mast cells produce LIF (12). Therefore, LAD2 cells were co-cultured with cytokine-free culture medium only, culture medium containing 100 ng/mL SCF, or decidualized or non-decidualized T HESCs. The LIF production level was then evaluated using an ELISA. The LIF production level of LAD2 cells after 100 ng/mL SCF administration was greater than after incubation with cytokine-free culture medium only (**Figure 2B**). When co-cultured with decidualized T HESCs, LIF production was greater than when co-cultured with cytokine-free culture medium only or non-decidualized T HESCs (**Figure 2B**).

### LIF Derived From LAD2 Cells Increases Migration and Extravillous Trophoblast (EVT) Differentiation, and Decreases Syncytiotrophoblast (ST) Differentiation of HTR-8/SVneo Cells

LIF induces the migration of trophoblasts (14); therefore, the effects of LIF produced from LAD2 co-cultured with T HESCs were evaluated. The migration of HTR-8/SVneo cells was comparable between culture medium only (as control) and non-decidualized or decidualized T HESCs without LAD2 (**Figure 3A**). The migration of HTR-8/SVneo cells was enhanced when non-decidualized or decidualized T HESCs were co-cultured with LAD2 cells compared with the control group (**Figure 3A**). This difference disappeared when anti-LIF neutralized antibody was administered (**Figure 3A**). The migration of HTR-8/SVneo cells was comparable between non-decidualized T HESCs co-cultured with LAD2 and decidualized T HESCs co-cultured with LAD2 (**Figure 3A**).

LIF also influences the differentiation of trophoblasts (14). A trophoblast differentiation marker was examined when LAD2 cells were co-cultured with decidualized T HESCs. The EVT differentiation marker, CD9 mRNA in HTR-8/SVneo cells was increased when co-cultured with culture supernatants of decidualized T HESCs and LAD2 cells compared with the culture supernatants of decidualized T HESCs without LAD2 cells (as control) (**Figure 3B**). This difference disappeared when anti-LIF neutralized antibody was administered (**Figure 3B**). Next, we analyzed the production of the β hCG ST differentiation marker of HTR-8/SVneo. FK is known to induce β hCG production from trophoblasts, and LIF is reported to inhibit this process (28). FK administration increased the production of β hCG from HTR-8/SVneo cells co-cultured with culture supernatants of decidualized T HESCs (as a control) (**Figure 3C**). The production of β hCG was decreased when co-cultured with culture supernatants of decidualized T HESCs co-cultured with LAD2 cells compared to culture supernatants from decidualized T HESCs following FK administration (**Figure 3C**). This difference disappeared following the administration of anti-LIF neutralized antibody (**Figure 3C**).

### Comparison of Mast Cell Numbers and SCF mRNA Between Histological Specimens From Uterine and Tubal Pregnancies

Next, the effects of decidual stromal cells on mast cell phenotype were evaluated *in vivo*. First, we compared the numbers of human mast cells in histological specimens between uterine and tubal pregnancies. Histological specimens from uterine pregnancies should include decidual stromal cells, while those from tubal pregnancy should be deficient in decidual stromal cells (29, 30). Uterine mast cells include three phenotypes (MCT-positive/MCC-positive, MCT-positive/MCC-negative, and MCT-negative/MCC-positive) (31). Decidual mast cells were counted using toluidine blue staining to detect all three mast cell phenotypes, while MCT immunostaining detected MCT-positive/MCC-positive and MCT-positive/MCC-negative mast cells. The number of toluidine blue-stained or MCT-positive mast cells in uterine pregnancy samples was greater than in tubal pregnancy samples (**Figures 4A, B**, representative photos in **Figure 5**). The numbers of toluidine blue-stained mast cells were comparable between MCT-positive mast cells in uterine and tubal pregnancies (**Figures 4A, B**, representative photos in **Figure 5**). The SCF mRNA levels in uterine pregnancy samples were greater than in tubal pregnancy samples (**Figure 4C**).

### Comparison of LIF mRNA-Positive Mast Cell and LIF mRNA-Positive Non-Mast Cell Numbers Between Histological Specimens From Uterine and Tubal Pregnancies

**Figure 6:**
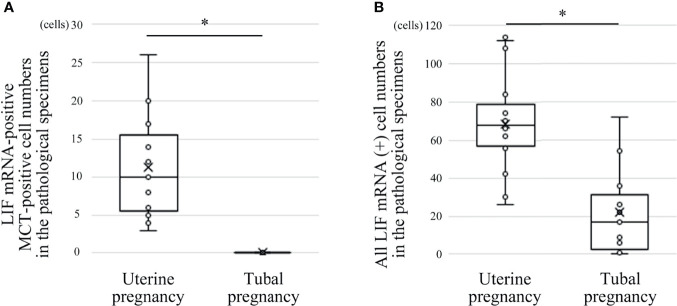
Comparison of LIF mRNA-positive mast cell and non-mast cell numbers between histological specimens from uterine and tubal pregnancies (19 cases of uterine pregnancy *vs.* 16 of tubal pregnancy). **(A)** LIF mRNA-positive MCT-positive cell numbers. *P < 0.05 compared with uterine pregnancy. **(B)** All LIF mRNA-positive cell numbers (including LIF mRNA-positive/MCT-positive and LIF mRNA-positive/MCT-negative cells). *P < 0.05 compared with uterine pregnancy.

**Figure 7:**
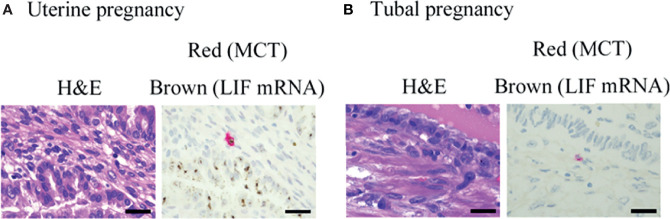
Representative photographs of mast cells in histological specimens from uterine and tubal pregnancy. LIF mRNA was detected by RNA scope (brown), and MCT protein was detected by immunohistochemistry (red) in the histological specimens. **(A)** Uterine pregnancy. Bars = 100 μM. **(B)** Tubal pregnancy. Bars = 100 μM.

Previously, we reported the histological expression of LIF mRNA in decidual mast cells (12). In the present study, the status of LIF mRNA in mast cells in histological specimens from uterine and tubal pregnancies was histologically evaluated. The LIF mRNA expression of mast cells in uterine pregnancy samples was retained, as previously reported (**Figure 6A**, and representative photo in **Figure 7A**); however, in tubal pregnancy, the expression was undetectable (**Figure 6A**, and representative photo in **Figure 7B**).

LIF mRNA is also expressed in non-mast immunocompetent cells, such as NK cells (17). The status of LIF mRNA was evaluated in non-mast immunocompetent cells and mast cells in histological specimens from uterine and tubal pregnancies. The number of LIF mRNA-positive cells in uterine pregnancy samples was greater than in tubal pregnancy samples (**Figure 6B**).

## Discussion

**Figure 8:**
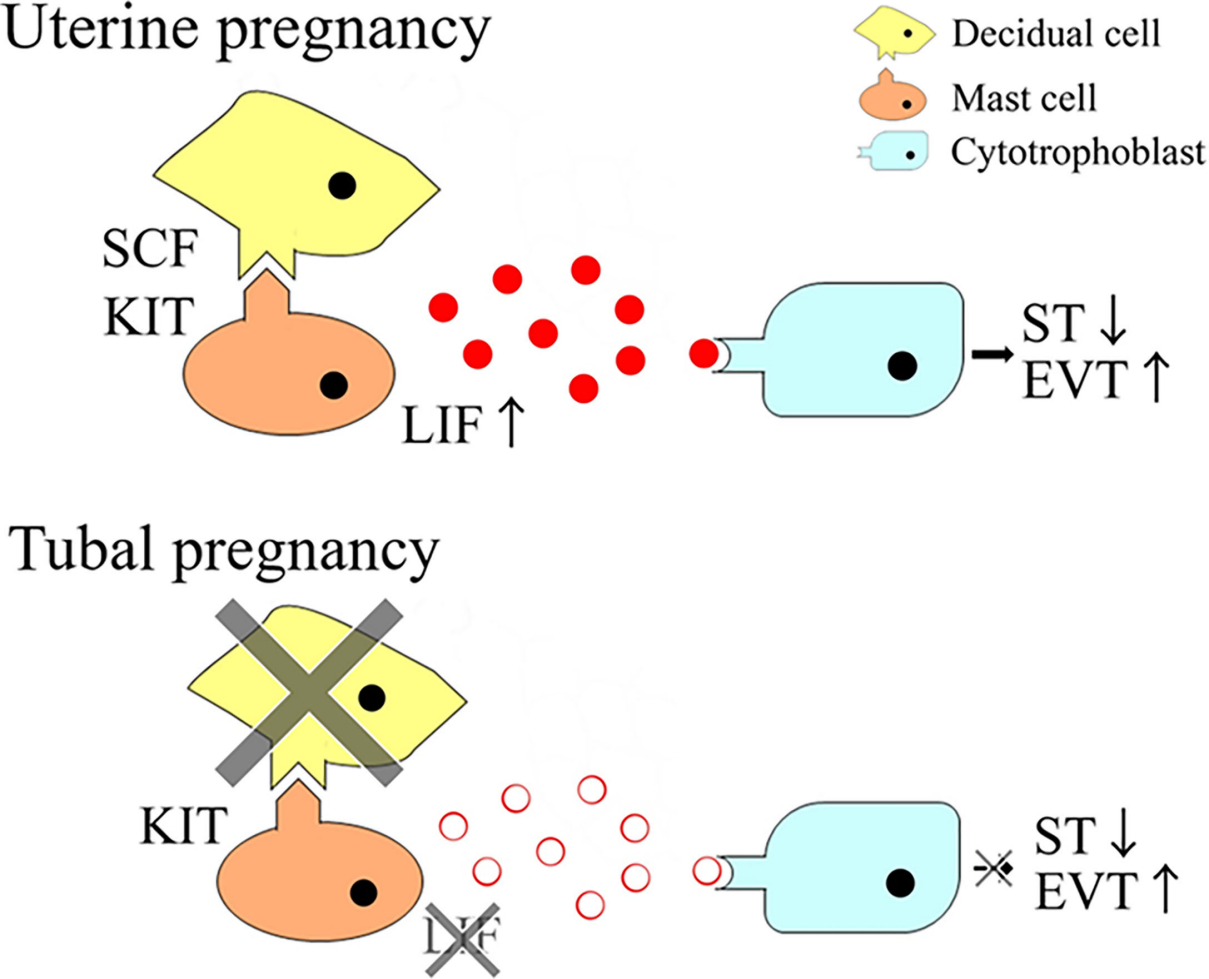
The current model.

The results of this study indicated that SCF derived from decidual stromal cells promoted the phenotype of human mast cells for successful pregnancy establishment (**Figure 8**).

This study showed the phenotypic differences of mast cells between uterine and tubal pregnancies. The number of mast cells in uterine pregnancies were greater than in tubal pregnancies. Additionally, mast cell LIF mRNA expression was detectable in uterine pregnancies but undetectable in mast cells from tubal pregnancies. Previously, we showed that KIR2DL4 was involved in the LIF production of mast cells (12). This study confirmed that mast cells in tubal pregnancies, and control tubes, expressed KIR2DL4 protein, similar to uterine pregnancies (as revealed by immunohistochemistry) (data not shown). Furthermore, KIR2DL4 did not seem to be involved in the phenotypic differences of mast cells between uterine and tubal pregnancies. Decidual stromal cells are known to be present during uterine pregnancy, but are absent in tubal pregnancies and control tubes (29, 30). Therefore, the association between mast cells and decidual stromal cells was investigated.

Decidualization increased the expression level of SCF mRNA in decidual stromal cells in the current *in vitro* study. A previous report also showed that decidual stromal cell expression increased the level of SCF mRNA more than endometrial stromal cells *in vivo* (32). SCF mRNA levels in the tissues were correlated with the number of mast cells in uterine and tubal pregnancies. This may be explained by the increase in mast cell migration caused by SCF (7). SCF induced LIF production in a human mast cell line of LAD2 cells. Therefore, SCF derived from decidual stromal cells seems to increase LIF mRNA levels in decidual mast cells during uterine compared to tubal pregnancies, which are deficient in decidual stromal cells. In placenta accreta samples, deeper areas where chorionic villi invade into the myometrium were deficient in decidual stromal cells, although decidual stromal cells remained in luminal areas. LIF mRNA was undetectable in mast cells in deeper areas of the placenta accreta, but remained in mast cells in luminal areas (**Supplementary Figure 1**). This observation also supports the presence of decidual stromal cells and the subsequent increase in SCF production associated with LIF mRNA expression in decidual mast cells.

LIF plays a role in trophoblast differentiation (18). The current data showed that LIF from LAD2 cells increased EVT differentiation and decreased ST differentiation of HTR-8/SVneo cells, which is compatible with a previous report (14). EVT invasion is an important step in spiral artery remodeling. Decidual mast cell-derived LIF would explain the important role of decidual mast cells in spiral artery remodeling in uterine pregnancy (6). In the present study, the abnormal trophoblast differentiation could not be detected in tubal pregnancies, although the number of LIF-positive mast cells was lower in tubal than uterine pregnancies. This can be explained by decidual NK cells compensating to produce sufficient levels of LIF. Decidual NK cells produce LIF and the number of NK and mast cells were reported to be counterbalanced in decidual tissues of mice (33). In humans, similar counterbalancing mechanisms could exist, although further studies are necessary to determine the molecular mechanism in tubal pregnancy.

LIF is known to enhance trophoblast invasion (14) and decidual stromal cells are also thought to enhance trophoblast invasion (24). Here, the migration of HTR-8/SVneo cells was comparable between non-decidualized T HESCs co-cultured with LAD2 and decidualized T HESCs co-cultured with LAD2. LIF production of LAD2 cells may be sufficient to induce HTR-8/SVneo invasion, even without decidual stromal cells.

This study was limited in that most of the results were based on *in vitro* experiments, and the *in vivo* data were based on a limited number of samples. Further studies will be required to confirm the findings.

## Data Availability Statement

The raw data supporting the conclusions of this article will be made available by the authors, without undue reservation.

## Ethics Statement

The studies involving human participants were reviewed and approved by The Ethical Research Committee of Iwate Medical University (MH2021-096). The patients/participants provided their written informed consent to participate in this study.

## Author Contributions

TK, TS, and HH conceived the project. TK and RS designed experiments. MO and SM collected the clinical samples. MO ad AS obtained the clinical data. CU, AU, and YS stained samples. CU, TK, HN, and RS performed *in vitro* experiments. CU performed statistical analysis. CU and TK compiled figures. TK wrote the manuscript. All authors reviewed the manuscript. All authors contributed to the article and approved the submitted version.

## Funding

This study was financially supported by JSPS KAKENHI (18K07014, 19K16556, and 21K06909).

## Conflict of Interest

The authors declare that the research was conducted in the absence of any commercial or financial relationships that could be construed as a potential conflict of interest.

## Publisher’s Note

All claims expressed in this article are solely those of the authors and do not necessarily represent those of their affiliated organizations, or those of the publisher, the editors and the reviewers. Any product that may be evaluated in this article, or claim that may be made by its manufacturer, is not guaranteed or endorsed by the publisher.

## Supplementary Material

Supplementary Figure 1 Representative photographs of mast cells in histological specimens from placenta accreta. LIF mRNA was detected by RNA scope (brown), and MCT protein was detected by immunohistochemistry (red). Bars = 100 μM.
