# Supplementary figures and images for: Decidualization of Stromal Cells Promotes Involvement of Mast Cells in Successful Human Pregnancy by Increasing Stem Cell Factor Expression
Source: Front Immunol. 2022 Jan 31;13:779574. doi: 10.3389/fimmu.2022.779574 (PMC8841734; doi:10.3389/fimmu.2022.779574)

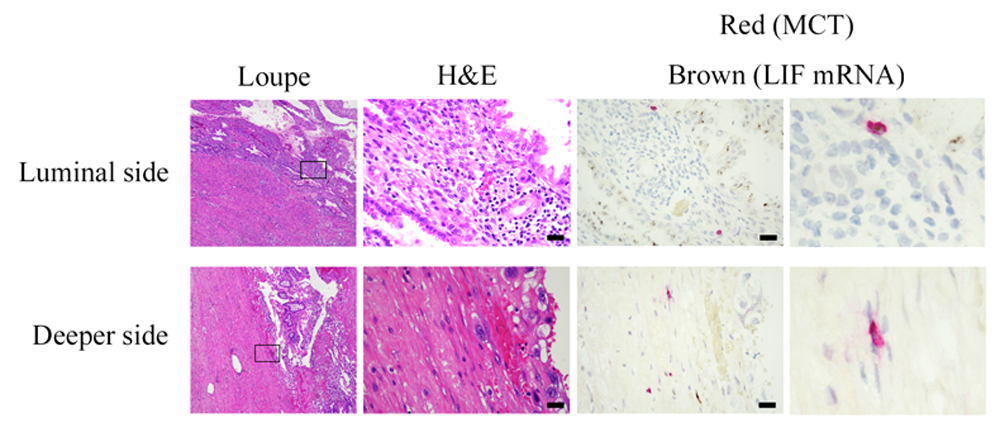

Supplement: Supplementary Figure 1 — Representative photographs of mast cells in histological specimens from placenta accreta. LIF mRNA was detected by RNA scope (brown), and MCT protein was detected by immunohistochemistry (red). Bars = 100 μM. [file Image_1.tif]
